# A combined miRNA–piRNA signature in the serum and urine of rabbits infected with *Toxoplasma gondii* oocysts

**DOI:** 10.1186/s13071-022-05620-0

**Published:** 2022-12-26

**Authors:** Shi-Chen Xie, Chun-Xue Zhou, Bin-Tao Zhai, Wen-Bin Zheng, Guo-Hua Liu, Xing-Quan Zhu

**Affiliations:** 1 Research Center for Parasites and Vectors, College of Veterinary Medicine, Hunan Agricultural University, Changsha, 410128 Hunan Province People’s Republic of China; 2 Laboratory of Parasitic Diseases, College of Veterinary Medicine, Shanxi Agricultural University, Taigu, 030801 Shanxi Province People’s Republic of China; 3 Department of Pathogen Biology, School of Basic Medical Sciences, Cheeloo College of Medicine, Shandong University, Jinan, 250012 Shandong Province People’s Republic of China; 4 Key Laboratory of Veterinary Pharmaceutical Development, Lanzhou Institute of Husbandry and Pharmaceutical Sciences, Chinese Academy of Agricultural Sciences, Ministry of Agriculture, Lanzhou, 730050 Gansu Province People’s Republic of China; 5 State Key Laboratory of Veterinary Etiological Biology, Key Laboratory of Veterinary Parasitology of Gansu Province, Lanzhou Veterinary Research Institute, Chinese Academy of Agricultural Sciences, Lanzhou, 730046 Gansu Province People’s Republic of China

**Keywords:** *Toxoplasma gondii*, Rabbit, Serum, Urine, miRNAs, piRNAs

## Abstract

**Background:**

Increasing evidence has shown that non-coding RNA (ncRNA) molecules play fundamental roles in cells, and many are stable in body fluids as circulating RNAs. Study on these ncRNAs will provide insights into toxoplasmosis pathophysiology and/or help reveal diagnostic biomarkers.

**Methods:**

We performed a high-throughput RNA-Seq study to comprehensively profile the microRNAs (miRNAs) and PIWI-interacting RNAs (piRNAs) in rabbit serum and urine after infection with *Toxoplasma gondii* oocysts during the whole infection process.

**Results:**

Total RNA extracted from serum and urine samples of acutely infected [8 days post-infection (DPI)], chronically infected (70 DPI) and uninfected rabbits were subjected to genome-wide small RNA sequencing. We identified 2089 miRNAs and 2224 novel piRNAs from the rabbit sera associated with *T. gondii* infection. Meanwhile, a total of 518 miRNAs and 4182 novel piRNAs were identified in the rabbit urine associated with *T. gondii* infection. Of these identified small ncRNAs, 1178 and 1317 serum miRNAs and 311 and 294 urine miRNAs were identified as differentially expressed (DE) miRNAs in the acute and chronic stages of infections, respectively. A total of 1748 and 1814 serum piRNAs and 597 and 708 urine piRNAs were found in the acute and chronic infection stages, respectively. Of these dysregulated ncRNAs, a total of 88 common DE miRNAs and 120 DE novel piRNAs were found in both serum and urine samples of infected rabbits.

**Conclusions:**

These findings provide valuable data for revealing the physiology of herbivore toxoplasmosis caused by oocyst infection. Circulating ncRNAs identified in this study are potential novel diagnostic biomarkers for the detection/diagnosis of toxoplasmosis in herbivorous animals.

**Graphical Abstract:**

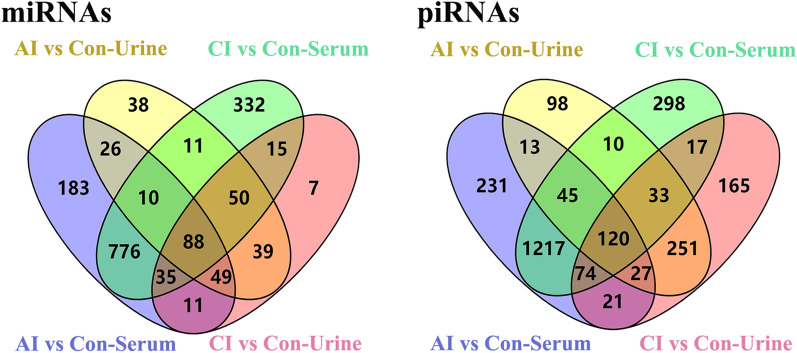

**Supplementary Information:**

The online version contains supplementary material available at 10.1186/s13071-022-05620-0.

## Introduction

*Toxoplasma gondii* is a eukaryotic parasite belonging to the phylum Apicomplexa [1]. It is an opportunistic pathogen of humans, infecting approximately one-fourth of the world’s population [2]. There are different local infection rates, and a few lineages predominate in different areas [3, 4]. Virulent *Toxoplasma* strains are usually lethal in a mouse model after inoculation with even a single parasite, whereas the LD_50_ of avirulent strains ranges from 10^3^ to 10^5^ in laboratory mice [5]. *Toxoplasma gondii* infection is usually asymptomatic in immunocompetent hosts, but can cause severe consequences in immune-compromised or pregnant hosts, leading to encephalitis, birth defects or even death [6]. In addition, studies in mice have indicated that *T. gondii* might be involved in manipulations of specific brain functions and behavioral responses [7, 8].

*Toxoplasma gondii* is an obligate intracellular parasite that has a broad host range, with cats and other feline animals as definitive hosts and all warm-blooded animals as intermediate hosts [9]. A recent review on *T. gondii* prevalence in meat animals worldwide indicated that the overall infection rates in sheep, pigs and cattle were 14.7%, 12.3% and 2.6%, respectively [10]. The European Food Safety Authority (EFSA) disclosed that approximately 60% of human toxoplasmosis cases result from the consumption of herbivore or omnivore meat [11, 12]. The environmentally resistant *T. gondii* oocysts are excreted within felid feces and remain viable in the sea water for > 1 year [13]. Sheep and goats are more susceptible to toxoplasmosis, and the main source of infection is *T. gondii* oocysts [14, 15]. Moreover, oocyst-induced infections are more severe and pathogenic than tissue cyst-induced toxoplasmosis [16]. However, herbivores showed a better resistance to infection of *T. gondii* than mice, and the mechanisms of resistance and immune induction in herbivores in response to infection of *T. gondii* oocysts remain unclear [17].

Small RNAs (sRNAs) are a class of single-stranded non-coding RNAs that are typically 18–30 nt in length and include microRNAs (miRNAs), small interfering RNAs (siRNAs), PIWI-interacting RNAs (piRNAs) and tRNA-derived fragments (tRFs) [18]. Since the first sRNA was identified in *Caenorhabditis elegans* in 1993, numerous sRNAs have been identified as the potential diagnostic biomarkers or as targets of therapeutics during disease progression by molecular biology and high-throughput detection methods [19, 20]. miRNAs are widely used as biomarkers for diagnosis or prognosis in a variety of diseases, because miRNAs can be stably present in cytoplasm and various body fluids and play an important role in cell differentiation, proliferation and survival [19, 21, 22]. In the development of parasitic diseases, numerous miRNAs and their biogenesis machinery have also been identified and characterized. For instance, Gputa et al. revealed that has-miR-3158-3p and has-miR-150-5p are potential biomarkers to distinguish between cerebral malaria patients and healthy people. In *T. gondii*-infected mice [23], the dysregulation of miR-146a and miR-155 expression is regarded as the significant signature in the host infected with type II strains of *T. gondii*, and these miRNAs have also been associated with establishment of chronic toxoplasmosis [24].

piRNAs are 24–31 nt in length and are another important class of noncoding RNAs involved in modulation of key signaling pathways at the transcriptional or post-transcriptional levels [25]. piRNAs associate with PIWI proteins to form a piRNA/PIWI complex, known as piRISC, thereby influencing transposon silencing to protect genome integrity [25]. piRNAs were first detected in mammalian germline and are now found expressed in a tissue-specific manner in many human tissues [26]. More evidence showed that piRNAs are abnormally expressed in various cancers [27, 28]. In addition, a growing number of studies have shown that some piRNAs stably occur in serum and their levels are correlated with the severity of illness [29–31]. More emerging studies have suggested that piRNAs may serve as novel diagnostic and prognostic biomarkers and therapeutic targets for some diseases [31, 32].

To investigate the potential roles of miRNAs and piRNAs during toxoplasmosis progression, we performed a high-throughput sequencing analysis to identify differentially expressed (DE) miRNAs and piRNAs in rabbit serum and urine following infection with *T. gondii* oocysts. This study presented the first transcriptome-wide ncRNA landscape in herbivore body fluids, which provides a valuable dataset that will facilitate the elucidation of the mechanisms underlying physiological changes during *T. gondii* infection.

## Methods

### Parasite and animals

Both Kunming mice (6–8 weeks old) and female Japanese white rabbits (3–4 months old) of specific pathogen-free (SPF) were purchased from the Laboratory Animal Center of Lanzhou Veterinary Research Institute, Chinese Academy of Agricultural Sciences (Lanzhou, Gansu Province, China). A domestic cat (Chinese Lihua breed, 3–4 months old) was purchased from Yantan pet market in Lanzhou city. All animals were raised in an environment with appropriate temperature and ventilation, 12 h of light and 12 h of darkness. Mice were given sterilized food and water ad libitum.

*Toxoplasma gondii* Prugniuad (Pru) strain (Type II) was kept in Kunming mice by oral inoculation with *T. gondii* cysts obtained from mice brain tissues 60 days post-infection (DPI). After anesthetizing, the brains of the infected mice were collected and homogenized in a sterile tissue homogenizer. The cysts were counted and diluted to 600 cysts/ml in phosphate-buffered saline solution (PBS).

### Animal infection and sample collection

A *T. gondii*-free cat was orally inoculated with 600 T*. gondii* cysts. The oocysts were isolated, purified and sporulated according to the methods previously described by Zhou et al. [33]. Subsequently, the viability and virulence of the obtained oocysts were confirmed using bioassays in mice (data not shown). After the collection of oocysts, the cat was killed by overdose of isoflurane. Twelve Japanese white rabbits were randomly assigned into three groups (*n* = 4 per group): acute infection (AI) group (8 DPI), chronic infection (CI) group (70 DPI) and control (Con) group. Rabbits in infection group were inoculated intragastrically with approximately 10^6^ oocysts suspended in 1 ml PBS. Control ones were gavaged with the same volume of PBS but without parasites. Infected rabbits showed typical signs of acute toxoplasmosis at 8 DPI, and blood and urine were obtained from the acutely infected rabbits. At 35 DPI, all infected animals returned to normal. Blood and urine were obtained from the chronically infected rabbits at 70 DPI. To remove cellular debris, urine samples were centrifuged at 3000 g for 10 min at 4 °C. Urine supernatants and sera were collected and frozen instantly in liquid nitrogen and stored at − 80 °C until further use.

### RNA isolation, library construction and sequencing

Total RNA was extracted from serum and urine samples of rabbits by the Trizol method (Invitrogen, Carlsbad, CA, USA) according to the manufacturer’s instructions. The quality and integrity of RNA samples were determined using the Agilent Bioanalyzer 2100 system (Agilent Technologies, CA, USA) and 1% agarose gel electrophoresis, respectively.

The small RNA library construction was performed according to the protocol previously described by Fehlmann et al. [34]. Briefly, sRNA fragments of 18–30 nt in length were separated and recycled by 15% urea-PAGE gel electrophoresis. sRNAs were ligated with a 5-adenylated, 3-blocked single-stranded DNA adapter. After RT primer hybridization, the 5′ adaptor was linked and the first strand cDNA was synthesized by a reverse transcription reaction. After several rounds of PCR amplification, 100–120 bp products were purified and obtained for BGISEQ-500 sequencing (BGI Inc.; Shenzhen, China).

### Analysis of RNA-Seq data

To obtain clean reads, raw reads were filtered by removing low-quality reads, reads containing poly-N, reads shorter than 18 nt, reads with 5′ adapter contaminants and reads without a 3′ adapter insert. All clean reads were aligned to *Oryctolagus cuniculus* genome and to other small ribonucleic acid databases (e.g. miRBase, Rfam, siRNA, piRNA and snoRNA) by Bowtie2 [35]. Then, mapped sRNAs were classified. To ensure every unique small RNA was mapped to only one category, the following priority rule was applied: MiRbase > piRNABank > snoRNA (plant) > Rfam > other sRNA. Meanwhile, miRDeep2 software (v0.1.2; https://github.com/rajewsky-lab/mirdeep2) was employed to predict novel miRNAs by exploring the characteristic hairpin structure of miRNA precursor, and Piano [36] was used to predict piRNAs. For target prediction, we applied multiple softwares, including RNAhybrid [37] and miRanda [38], to find potential targets of miRNAs. DEseq2 R package was used to perform differential expression analysis [39]. The *P*-values were adjusted to *Q* values for multiple testing corrections employing the Benjamini and Hochberg method. Corrected *P*-value < 0.05 and the absolute value of Log_2_ fold-change above 1 were set as the threshold for significantly differential expression by default [40].

## Results

### Characteristics of infected animals

**Fig. 1:**
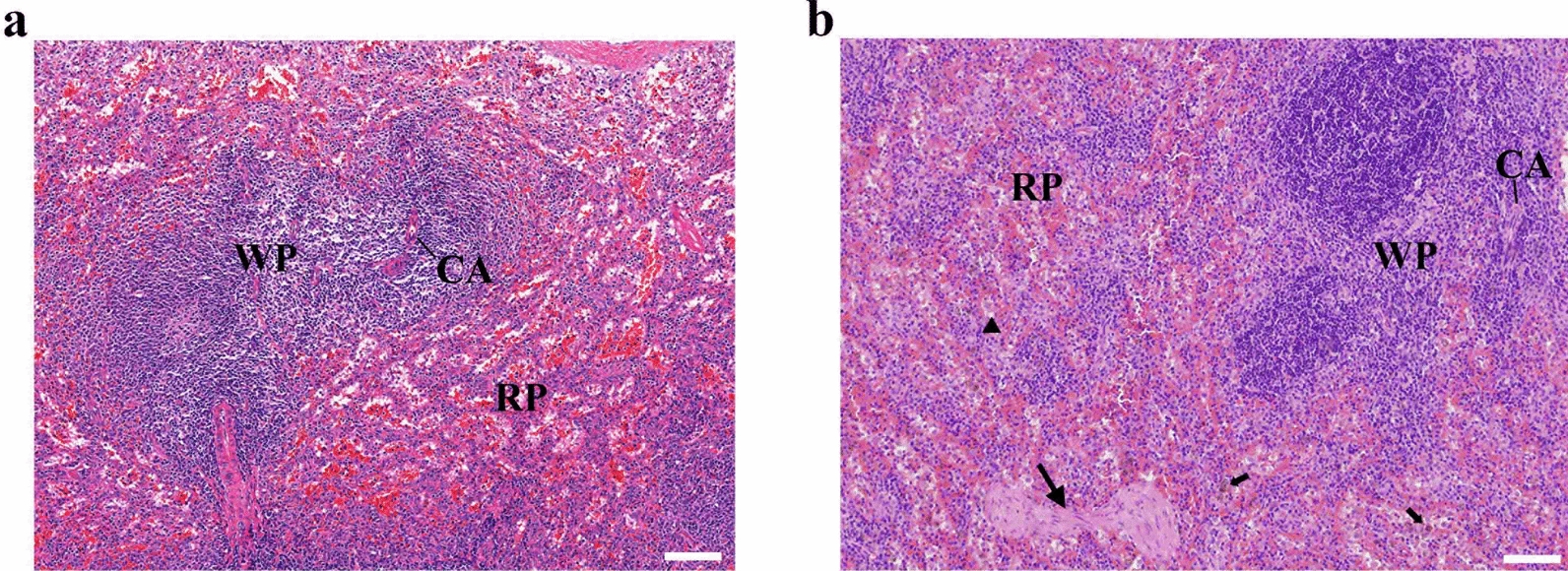
Histological features of spleen section from healthy control rabbits and rabbits experimentally infected with *Toxoplasma gondii*. Images showing the H&E-stained spleen section at 100× (**a**, **b**). **a** Spleen section from healthy, uninfected rabbit. The structures of white pulp (WP) and red pulp (RP) were clearly identified with normal cell density. **b** Spleen section from a rabbit with acute *T. gondii* infection. The number and dimension of splenic nodule are increased, and more plasma cells are observed in the splenic cord (black triangle) of red pulp. Note that granulomas are present (big black arrow). Hemosiderin deposition (small black arrow) indicated red blood cell destruction. *CA* central arteriole. Scale bar = 100 μm

Rabbits were examined daily for clinical signs, including body weight, body temperature and food intake, until 35 DPI, and the experiment was ended at 70 DPI. In this study, infected rabbits showed typical acute signs of toxoplasmosis from 5 DPI, and peaked at 8 DPI, including high temperature (> 40 °C), loss of appetite, depression and lethargy. Histopathological changes in spleen were characterized by the breakdown of the structure, the reduction of the white pulp and the increase in the number of megakaryocytes (Fig. 1). All infected rabbits survived and their physical status was restored at 35 DPI. No clinical symptoms were observed in the healthy control rabbits throughout the whole experiment.

### Expression of small RNAs in serum from both *Toxoplasma*-infected and normal rabbits

**Fig. 2:**
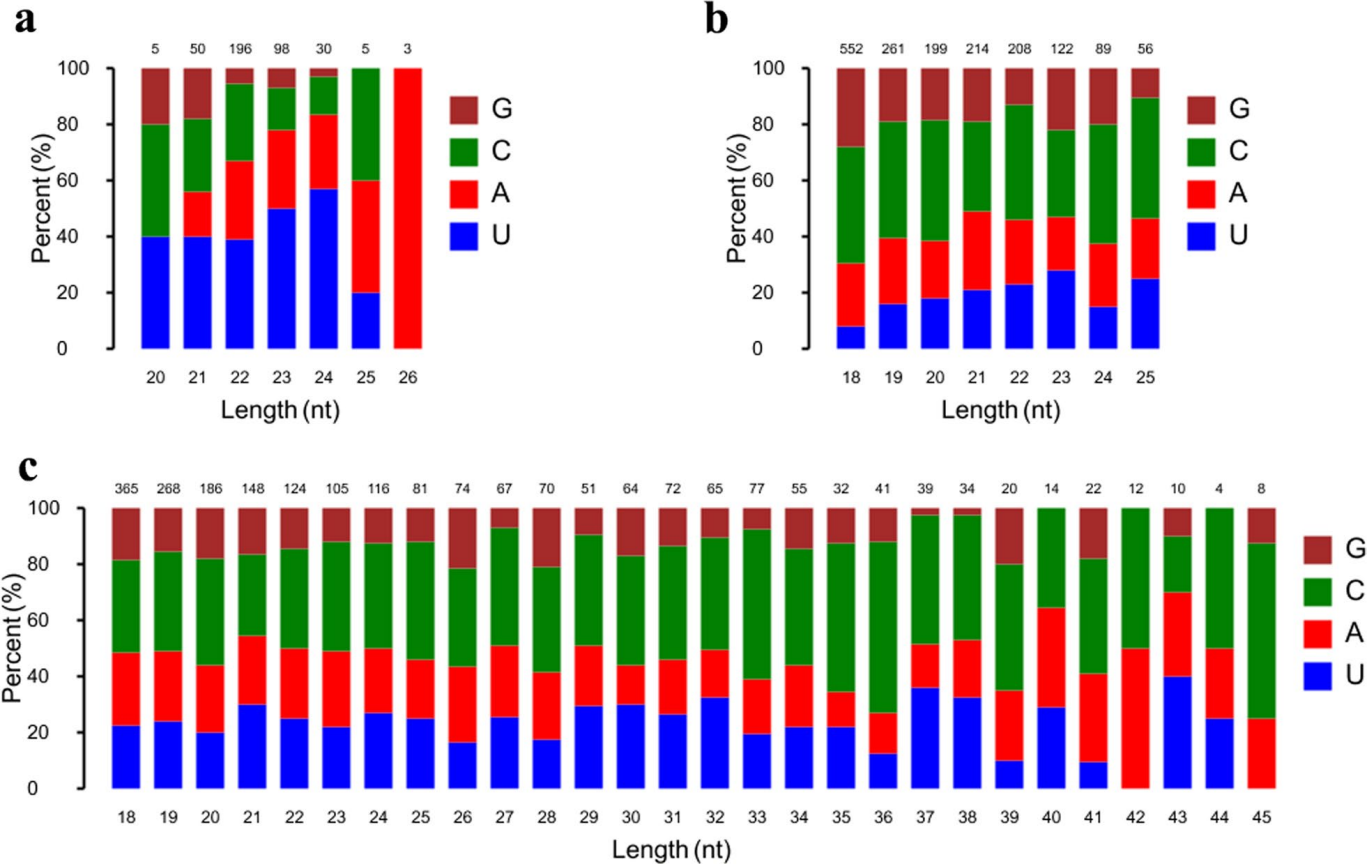
First nucleotide bias of obtained small RNA in serum samples of rabbits. **a** First nucleotide bias of known miRNAs in rabbit serum. **b** First nucleotide bias of predicted miRNAs in rabbit serum. **c** First nucleotide bias of predicted piRNAs in rabbit serum

**Fig. 3:**
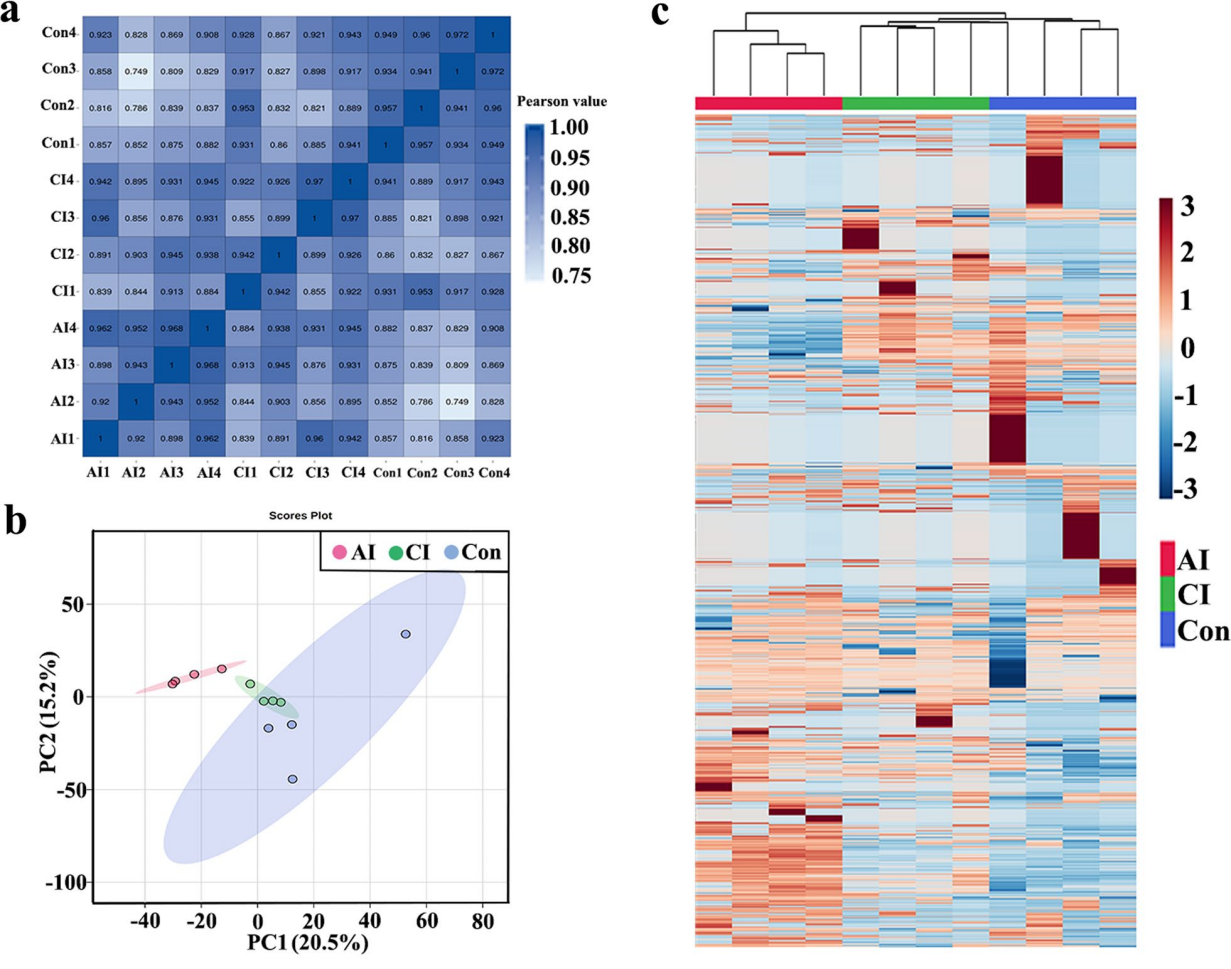
Global view of temporal sRNA expression profiles in rabbit serum during *T. gondii* infection. **a** The sRNA correlation heatmap of sample clustering. **b** Principal component analysis of all identified serum sRNA. **c** Unsupervised hierarchical clustering of sRNA profiling data. sRNA intensity is normalized so that blue represents low intensity and yellow represents high intensity. Columns are hierarchically clustered based on a complete linkage using Pearson correlation coefficients as the distance measure. Sample groups including acutely infected rabbits, chronically infected rabbits and uninfected control rabbits are labeled as AI, CI and Con, respectively

To explore small RNA profiles in serum and urine during toxoplasmosis progression, RNA sequencing was used to analyze sRNA expression in serum and urine from oocysts-infected and non-infected rabbits. After removing low-quality reads, > 19.78 million high-quality clean reads were obtained. The read quality is shown in Additional file 1: Table S1. Of the filtered clean reads, 76.50–91.50% detected in serum were mapped to the reference *Oryctolagus cuniculus* genome. The first residue at the 5′ terminus of the 20–26 nt known miRNAs is predominantly uridine (U) (Fig. 2a), while the first residue at the 5′ terminus of the predicted miRNAs is predominantly cytosine (C) (Fig. 2b). As shown in Fig. 2c, the first residue at the 5′ terminus of the 18–45 nt predicted piRNAs is predominantly cytosine (C). To explore the similarity of serum samples, we performed Pearson correlation analysis, principal component analysis (PCA) and hierarchical clustering analysis. As shown in Fig. 3a, a Pearson correlation matrix separated the AI samples from the other samples, as illustrated by the heatmap correlation matrices (Spearman correlations [*r*] = 0.898–0.968), showing a high level of correlation within Con samples ([*r*] = 0.934–0.972). As shown in Fig. 3b, PCA score plots did not clearly differentiate chronically infected rabbits from uninfected control rabbits. However, hierarchical clustering displayed that the sRNA expression pattern in acutely infected rabbits was distinguishable compared to that of chronically infected rabbits and uninfected control rabbits (Fig. 3c).

### Profiling of small RNAs in rabbit urine during toxoplasmosis progression

**Fig. 4:**
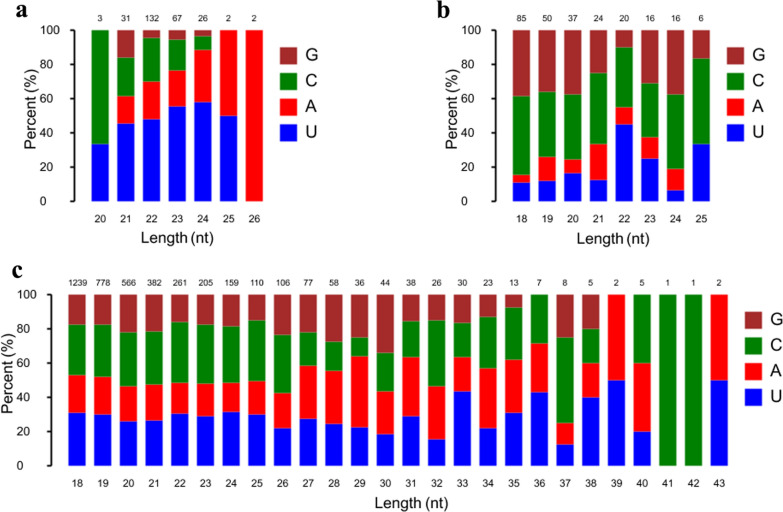
First nucleotide bias of obtained small RNA in urine samples of rabbits. **a** First nucleotide bias of known miRNAs in rabbit urine. **b** First nucleotide bias of predicted miRNAs in rabbit urine. **c** First nucleotide bias of predicted piRNAs in rabbit urine

**Fig. 5:**
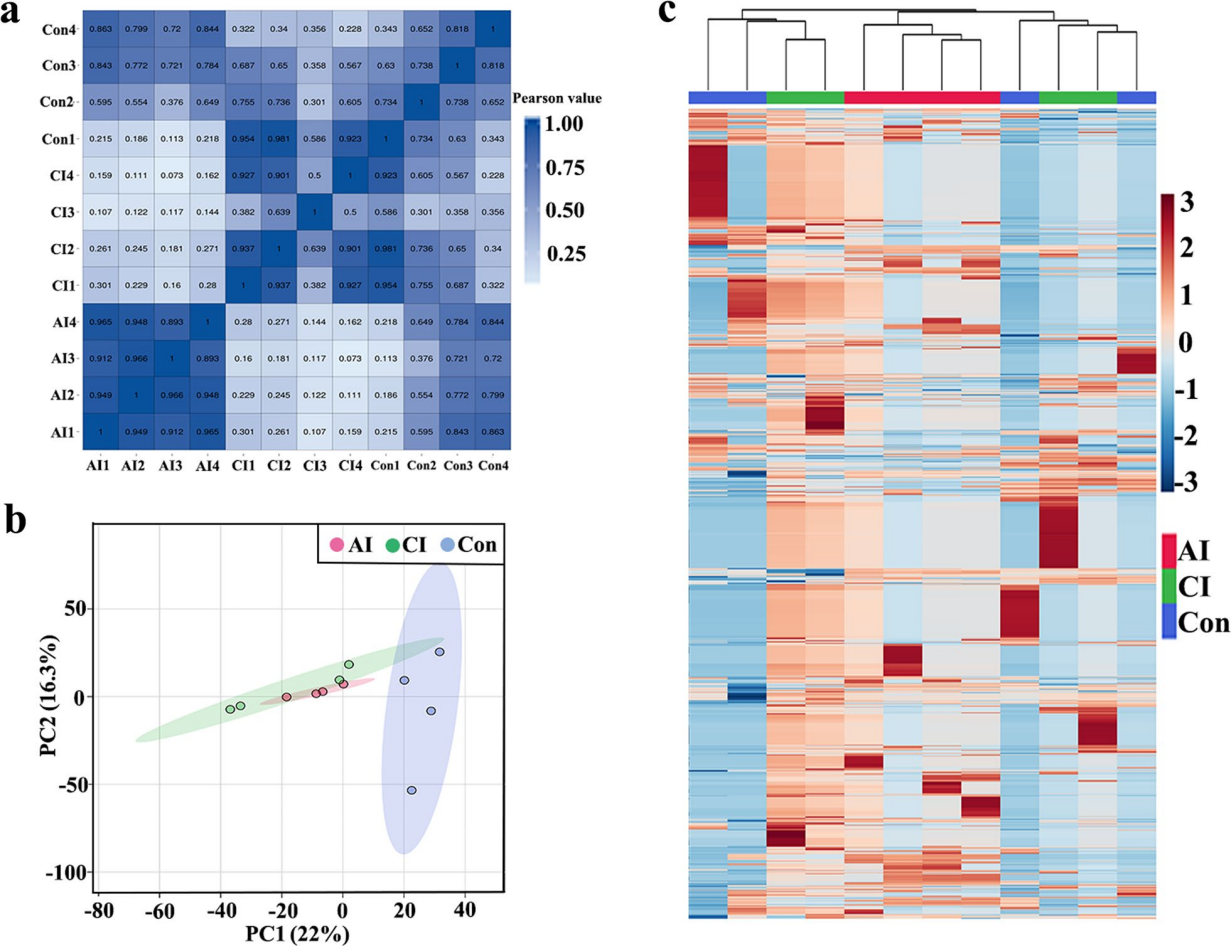
Global view of temporal sRNA expression profiles in rabbit urine during *T. gondii* infection. **a** The sRNA correlation heatmap of sample clustering. **b** Principal component analysis of all identified urine sRNAs. **c** Unsupervised hierarchical clustering of sRNA profiling data. sRNA intensity is normalized so that blue represents low intensity and yellow represents high intensity. Columns were hierarchically clustered based on a complete linkage using Pearson correlation coefficients as the distance measure. Sample groups are acutely infected rabbits, chronically infected rabbits and uninfected control rabbits, which are labeled as AI, CI and Con, respectively

In this study, > 19.77 million high-quality clean reads in each library were obtained and used for subsequently analysis. The sequencing data are summarized in Additional file 2: Table S2. As shown in Fig. 4a, the percent of cytosine (C) at the 5′ terminus of the 20–26 nt known miRNAs decreases with the increase of the miRNA length. However, the percent of adenine (A) increased with the increase of the miRNA length. The majority of the first residue at the 5′ terminus of the 20–26 nt predicted miRNAs is cytosine (C) (Fig. 4b). As shown in Fig. 4c, the number of piRNA decreased with the increase of the piRNA length and the first residue at the 5′ terminus of the 18–26 nt predicted piRNAs is predominantly cytosine (C). As shown in Fig. 5a, a Pearson correlation matrix separated the AI samples from the other samples, as illustrated by the correlation matrices (Spearman correlations [*r*] = 0.893–0.966). As shown in Fig. 5b, PCA score plots clearly distinguish acutely infected rabbits from uninfected control rabbits, but samples from acutely infected and chronically infected rabbits overlapped. Meanwhile, heatmaps revealed that the sRNA expression pattern in acutely infected rabbits is distinct from that of chronically infected rabbits and uninfected control rabbits (Fig. 5c).

### DE miRNAs and DE piRNAs in serum and urine following *T. gondii* infection

**Fig. 6:**
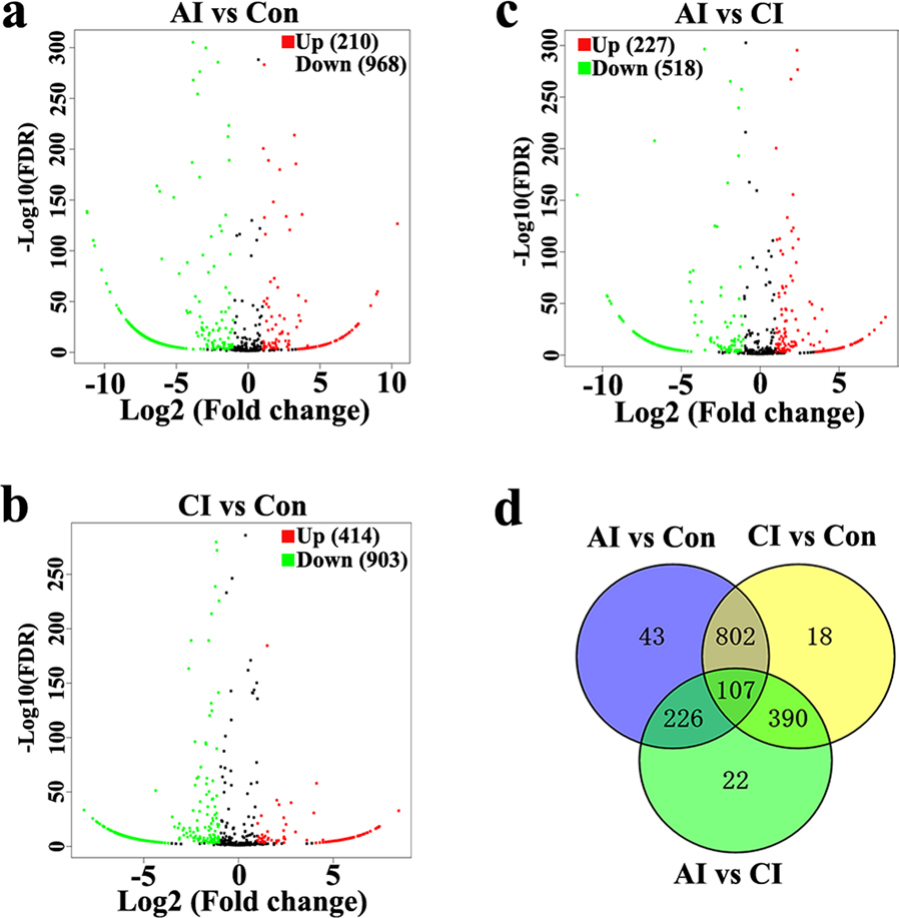
Serum miRNA profile analysis among different groups. **a** The volcano plot shows the individual statistically significant miRNA between acutely infected group and control group. In this plot, the *x*-axis is log2 fold-change, which shows the direction of the change (negative scale is decrease and positive scale is increase) in the levels of miRNA expression, while the *y*-axis is the –log10 FDR, which shows the significance of the change. **b** The volcano plot shows the individual statistically significant miRNA between chronically infected rabbits and control rabbits. **c** The volcano plot shows the individual statistically significant miRNA between acutely infected rabbits and chronically infected rabbits. **d** Venn diagram shows number of differentially expressed miRNA among different comparison pairs. FDR represents false discovery rate

**Fig. 7:**
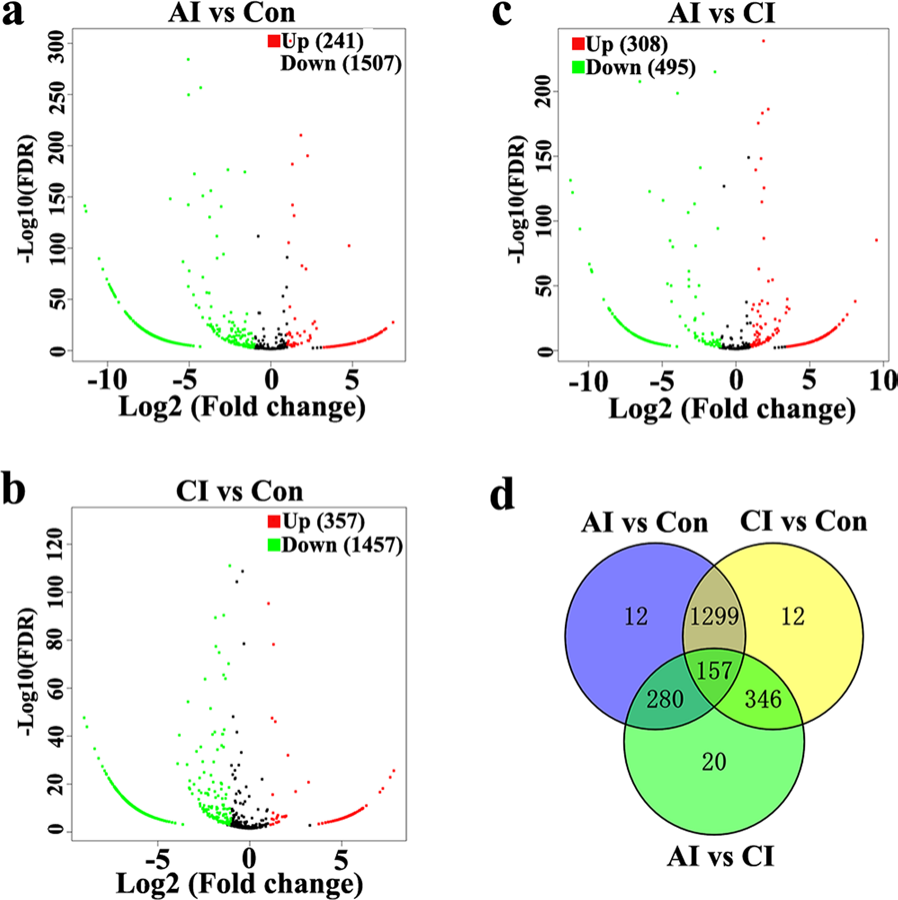
Serum piRNA profile analysis among different groups. **a** The volcano plot shows the individual statistically significant piRNA between acutely infected rabbits and control rabbits. In this plot, the *x*-axis is log2 fold-change, which shows the direction of the change (negative scale is decrease and positive scale is increase) in the levels of piRNA expression, while the *y*-axis is the –log10 FDR, which shows the significance of the change. **b** The volcano plot shows the individual statistically significant piRNA between chronically infected rabbits and control rabbits. **c** The volcano plot shows the individual statistically significant piRNA between acutely infected rabbits and chronically infected rabbits. **d** Venn diagram shows number of differentially expressed piRNA among different comparison pairs. FDR represents false discovery rate

**Fig. 8:**
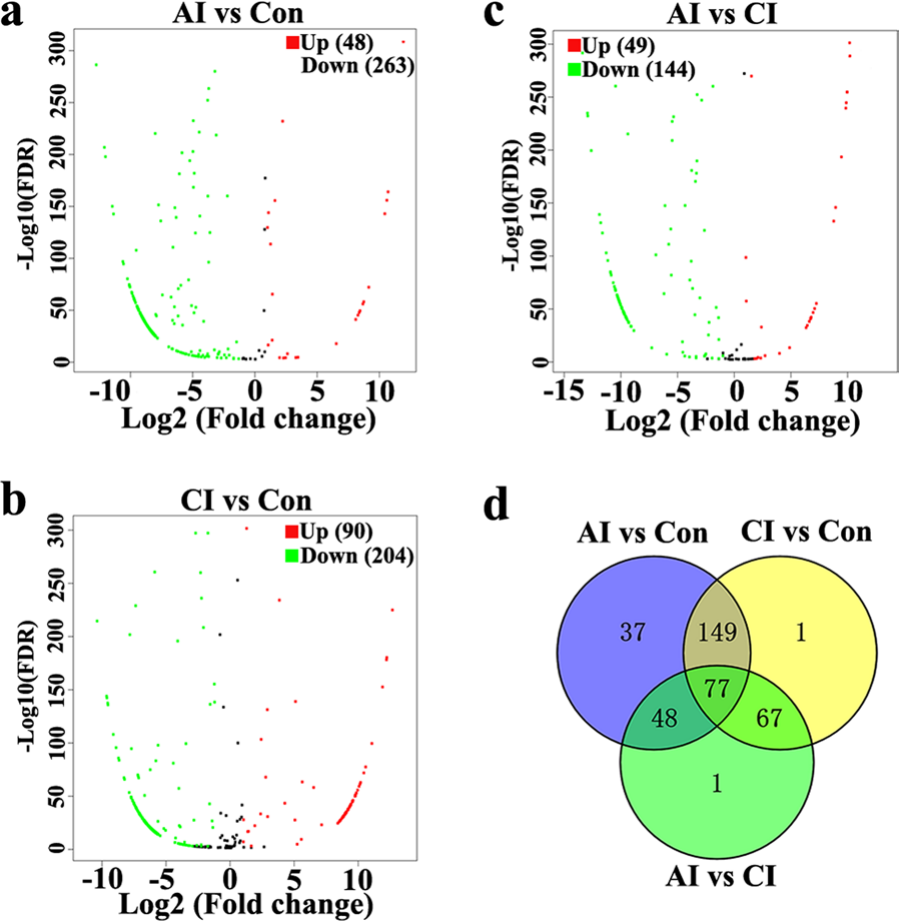
Urine miRNA profile analysis among different groups. **a** The volcano plot shows the individual statistically significant miRNA between acutely infected rabbits and control rabbits. In this plot, the *x*-axis is log2 fold-change, which shows the direction of the change (negative scale is decrease and positive scale is increase) in the levels of miRNA expression, while the *y*-axis is the –log10 FDR, which shows the significance of the change. **b** The volcano plot shows the individual statistically significant miRNA between chronically infected rabbits and control rabbits. **c** The volcano plot shows the individual statistically significant miRNA between acutely infected rabbits and chronically infected rabbits. **d** Venn diagram shows number of differentially expressed miRNA among different comparison pairs

**Fig. 9:**
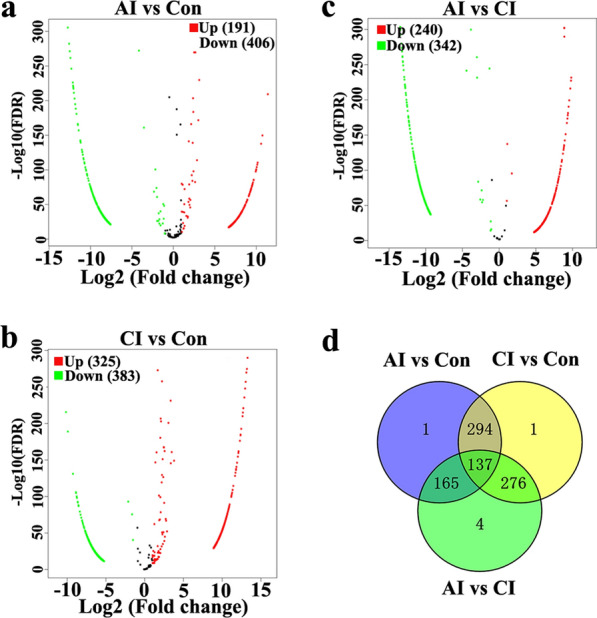
Urine piRNA profile analysis among different groups. **a** The volcano plot shows the individual statistically significant piRNA between acutely infected group and control group. In this plot, the *x*-axis is log2 fold-change, which shows the direction of the change (negative scale is decrease and positive scale is increase) in the levels of piRNA expression, while the *y*-axis is the –log10 FDR, which shows the significance of the change. **b** The volcano plot shows the individual statistically significant piRNA between chronically infected group and control group. **c** The volcano plot shows the individual statistically significant piRNA between acutely infected group and chronically infected group. **d** Venn diagram shows number of differentially expressed piRNA among different comparison pairs

Then, the volcano plots were used to analyze DE miRNAs and DE piRNAs in serum and urine from uninfected, acutely infected and chronically infected rabbits. In serum, 210 upregulated and 968 downregulated miRNAs were identified between acutely infected rabbits and control groups (Fig. 6a). Comparison between chronically infected and control rabbits showed more dysregulated miRNAs, including 414 upregulated and 903 downregulated miRNAs (Fig. 6b). As shown in Fig. 6d, 107 miRNAs were shared among the three comparison groups. Compared to the control group, the majority of DE piRNAs were common in both the acute and chronic infection groups. More DE piRNAs (*n* = 1814) were found in CI versus Con (Fig. 7d). Compared with serum, there are fewer DE miRNAs identified in urine. As shown in Fig. 8a, b, there are 311 and 294 DE miRNAs in AI versus Con and CI versus Con, respectively. Moreover, there are 226 common DE miRNAs in both AI versus Con and CI versus Con (Fig. 8d). Meanwhile, there are 597 and 708 DE piRNAs in AI versus Con and CI versus Con, respectively (Fig. 9a, b). There are 431 common DE piRNAs in both AI versus Con and CI versus Con (Fig. 9d).

**Fig. 10:**
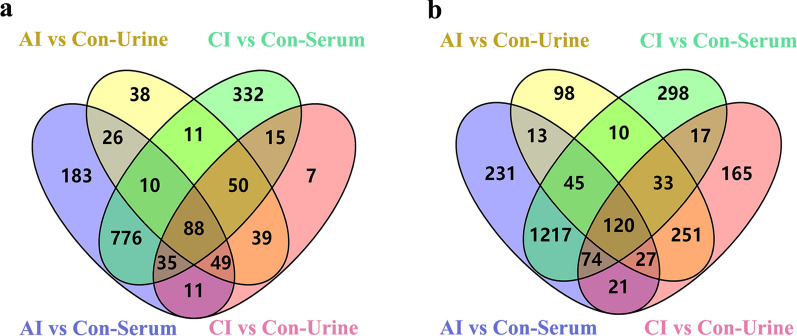
Venn diagrams showing the common and unique DE miRNAs (**a**) and DE piRNAs (**b**) in both serum and urine between the acutely and chronically infected rabbits *versus* uninfected rabbits

Furthermore, there are 88 common DE miRNAs found in both serum and urine samples of infected rabbits, which include 20 known miRNAs and 68 novel miRNAs (Fig. 10a). A complete list of the above-mentioned DE miRNAs is available in Additional file 3: Table S3. It is worth mentioning that the majority of the common DE miRNA were downregulated in all infected sera (78/88) and infected urine samples (73/88). However, two miRNAs (miR-138-5p and miR-509c-5p) in infected sera and miR-191-3p in urine were up-expressed during the whole toxoplasmosis progression. As shown in Fig. 10b, a total of 120 DE novel piRNAs were identified in all infected serum and urine samples, of which 110 piRNAs were downregulated in infected sera and 98 piRNAs were downregulated in the infected urine. Detailed information of the common DE piRNAs are shown in Additional file 4: Table S4.

## Discussion

Alterations in tissue or intracellular small RNA profiles in response to *Toxoplasma* infection have been previously reported [41–43]. These small RNAs may be involved in the regulation of parasite infection, mediation of protozoa-induced pathogenesis or assistance of host response against infection. Recently, more evidence has demonstrated that small RNAs extracellularly in various body fluids are numerous and act as a mirror of ongoing cellular and organ system functions, which indicates that small RNAs in body fluids can potentially reflect some physiopathologic changes [22, 44]. In addition, small RNAs in body fluids have been found to be stable and readily detectable and become attractive candidates for diagnosis, prognosis and targets for therapy in various diseases [22, 45]. However, the origin and function of these circulating small RNAs remain a mystery. The main goal of the present study was to explore the circulating small RNAs (mainly miRNAs and piRNAs) during *Toxoplasma* infection in rabbits and correlate them with mechanisms of *Toxoplasma*-induced pathogenesis.

Body fluids, especially blood and urine, are a rich resource for potential diagnostic biomarkers for a number of diseases. In the present study, we used a high-throughput RNA-Seq methodology to comprehensively profile the miRNAs and piRNAs in rabbit serum and urine after infection with *T. gondii* oocysts. We identified 2089 miRNAs and 2224 novel piRNAs from the rabbit sera associated with *T. gondii* infection. Meanwhile, a total of 518 miRNAs and 4182 novel piRNAs were identified in the rabbit urine. Of these identified small RNAs, a significant change of 1178 and 1317 serum miRNAs and 311 and 294 urine miRNAs were revealed in the acute and chronic infections, respectively. At the same time, a total of 1748 and 1814 serum piRNAs and 597 and 708 urine piRNAs were found in the acute and chronic infections, respectively. This is the first study of small RNAs in the herbivore biofluids during *T. gondii* infection, which provided valuable data for further development of molecular diagnostics for *T. gondii* infection in herbivorous animals.

miRNAs are regulatory components that can modulate gene expression at the post-transcriptional level and inhibit mRNA translation into proteins [19]. In addition to numerous studies on miRNA regulations in cancers [46], many miRNAs are differentially expressed in protozoal diseases, such as cryptosporidiosis [47], trypanosomosis [48], leishmaniasis [49] and toxoplasmosis [41, 43, 50]. Previous studies mainly focused on miRNA changes in cyst-induced toxoplasmosis. For instance, altered expression of miR-712-3p, miR-217-5p and miR-511-5p were detected in murine plasma, and their *Toxoplasma* infection specificity made them have a potential as biomarkers for diagnosis as well [51]. Additionally, the infection by *Toxoplasma* cysts leads to altered miRNA expression in mouse brain and spleen [52, 53]. Later, Hu et al. [43] revealed 38 DE miRNAs in chronically infected mouse brain following oocyst infection, which indicates that miRNAs might be involved in murine cerebral toxoplasmosis. However, it is unknown whether *Toxoplasma* infection could regulate host circulating miRNAs during *T. gondii* infection, especially in herbivores.

In this study, a total of 88 common DE miRNAs were identified in both rabbit serum and urine after infection with *T. gondii* oocysts at both acute and chronic infection stages. Among them, Ocu-miR-31-5p is one of the most significantly downregulated known miRNAs in infected serum during the whole toxoplasmosis progression. miR-31-5p is found upregulated in a number of tumors and is involved in cell cycle progression and DNA repair [54, 55]. However, a previous study reported that miR-31-5p was downexpressed in the chronic diabetic wounds compared with nondiabetic wounds, and it promoted angiogenesis, fibrogenesis and reepithelization [56]. The biological roles of miR-31-5p in toxoplasmosis still need further exploration. Ocu-miR-155-5p is another down-expressed miRNA in both rabbit urine and serum during the whole infection process. miR-155 is commonly expressed in different types of tissues and cells and responds quickly to infection and inflammatory stimuli and play a critical role in differentiation of macrophages into different phenotypes [57, 58]. In *T. gondii*-infected patients, miR-155-5p was up-expressed in ocular toxoplasmosis patients than asymptomatic individuals [59]. In another study, Zhou et al. found that miR-155-5p was upregulated in brain tissue in mice infected with cysts or oocysts [41]. The inconsistency with findings in the present study might result from the species variation and different tissue types. In a previous study, miR-326-3p was found downregulated in oocyst-infected mouse brains at chronic infection stage [43], which is downexpressed in both rabbit serum and urine during the whole infection. Interestingly, miR-326-3p is involved in neurological diseases, central nervous system development and cell morphology [60]. Serum miR-138-5p was significantly upregulated at both chronic and acute infection stages. However, miR-138-5p showed a diminished trend in a series of cancers, such as colorectal and pancreatic cancer [61, 62]. Restored expression of miR-138-5p suppresses autophagy in pancreatic cancer by targeting SIRT1 and thus suppresses pancreatic cancer cell proliferation [62]. A recent study showed that miR-138-5p was downregulated in HBV-positive HCC tissues and cells and hindered HBV replication and expression via targeting TNFAIP3 [63].

piRNAs are a novel class of non-coding RNAs. In addition to being widely present in various tissues, piRNAs have been proved to be stably present in body fluids, including the serum and urine, in a very stable form [44]. In this study, 1748 and 1814 DE novel piRNAs were found in rabbit serum at acute infection stage and chronic stage, respectively. In urine, there are 597 and 708 DE piRNAs in AI versus Con and CI versus Con, respectively. A total of 120 DE novel piRNAs were found to be significantly dysregulated not only within the serum but also in the urine. However, the roles of those DE piRNAs identified in this study need to be elucidated in further studies.

miRNAs are relatively stable and highly conserved across different species, which can be released from cell cytoplasm to extracellular fluids by forming vesicles [22]. By contrast, it remains unclear how piRNAs are generated in mammals. In this study, we found that the profile of miRNAs and piRNAs in rabbit serum and urine changed during *T. gondii* infection and identified many dysregulated miRNAs and piRNAs as promising biomarkers for the diagnosis of *Toxoplasma* infection in herbivorous animals. Further studies are warranted to reveal the roles of these dysregulated extracellular miRNAs and piRNAs in the pathological process of *Toxoplasma* infection.

## Conclusion

Collectively, to our knowledge this is the first study of small RNA profiling in rabbit serum and urine after infection with *Toxoplasma* oocysts, which reveals that *Toxoplasma* oocyst infection leads to alterations in the expression of miRNAs and piRNAs in the serum and urine of the herbivores. The findings of the present study provide potential biomarkers for the development of molecular tools for detection/diagnosis of *T. gondii* infection and also provide novel resources to further elucidate the exact roles of these small RNAs in the course of *Toxoplasma* infection.

## Supplementary Information

**Additional file 1. Table S1**: The list of small RNA sequencing data from serum in the present study.

**Additional file 2. Table S2**: The list of small RNA sequencing data from urine in the present study.

**Additional file 3. Table S3**: Common dysregulated microRNAs detected in both infected serum and urine samples.

**Additional file 4. Table S4**: Common dysregulated piRNAs detected in both infected serum and urine samples.

## Data Availability

The RNA-seq raw data described in the present study have been submitted to the NCBI Short Read Archive database (https://www.ncbi.nlm.nih.gov/sra) under the Sequence Read Archive (SRA) submission numbers PRJNA895697 and PRJNA895700. The full dataset is also available from Chun-Xue Zhou upon request (zhouchunxue23@163.com).

## Abbreviations

ncRNA: Non-coding RNA
sRNA: Small RNA
siRNA: Small interfering RNA
piRNA: PIWI-interacting RNA
miRNA: MicroRNA
tRFs: TRNA-derived fragments
DPI: Days post-infection
SPF: Specific pathogen free
DE: Differentially expressed
EFSA: The European Food Safety Authority
tRFs: TRNA derived fragments
Pru: Prugniuad
PBS: Phosphate-buffered saline solution
PCA: Principal component analysis
AI: Acute infection
CI: Chronic infection
Con: Control
QC: Quality control
