# Supplementary material for: A combined miRNA–piRNA signature in the serum and urine of rabbits infected with Toxoplasma gondii oocysts
Source: Parasit Vectors. 2022 Dec 26;15:490. doi: 10.1186/s13071-022-05620-0 (PMC9793633; doi:10.1186/s13071-022-05620-0)
Supplement: Supplementary file 1 — Additional file 1. Table S1: The list of small RNA sequencing data from serum in the present study. [file 13071_2022_5620_MOESM1_ESM.docx]

**Additional file 1: Table S1.** The list of small RNA sequencing data from serum in the present study.

| Library | Raw tag count | Low quality tag count | Invalid adapter tag count | PolyA tag count | Short valid length tag | Clean tag count | Q20 of clean tag (%) |
| --- | --- | --- | --- | --- | --- | --- | --- |
| Con-1 | 31,578,947 | 610,561 | 721,521 | 124 | 6,325,581 | 23,921,160 | 99.4 |
| Con-2 | 33,333,333 | 565,750 | 1,055,137 | 242 | 7,863,669 | 23,848,535 | 99.5 |
| Con-3 | 24,225,991 | 507,742 | 535,449 | 295 | 3,395,157 | 19,787,348 | 99.1 |
| Con-4 | 28,235,294 | 697,832 | 522,278 | 363 | 2,909,606 | 24,105,215 | 99.1 |
| AI-1 | 32,876,712 | 711,938 | 1,733,350 | 59 | 6,406,224 | 24,025,141 | 99.2 |
| AI-2 | 31,578,947 | 723,673 | 910,399 | 106 | 5,980,811 | 23,963,958 | 99.4 |
| AI-3 | 30,769,230 | 699,345 | 1,610,242 | 174 | 4,501,888 | 23,957,581 | 99.1 |
| AI-4 | 30,000,000 | 728,041 | 559,943 | 65 | 4,772,573 | 23,939,378 | 99.1 |
| CI-1 | 36,363,636 | 939,588 | 948,771 | 82 | 10,570,703 | 23,904,492 | 99.1 |
| CI-2 | 30,379,746 | 643,021 | 735,905 | 150 | 5,077,800 | 23,922,870 | 99.2 |
| CI-3 | 29,995,752 | 568,755 | 501,928 | 92 | 2,189,071 | 26,735,906 | 99.1 |
| CI-4 | 31,168,831 | 717,463 | 916,111 | 167 | 5,600,993 | 23,934,097 | 99.1 |

Abbreviations: Con, control group; AI, acutely infected group; CI, chronically infected group.
