# Supplementary material for: A combined miRNA–piRNA signature in the serum and urine of rabbits infected with Toxoplasma gondii oocysts
Source: Parasit Vectors. 2022 Dec 26;15:490. doi: 10.1186/s13071-022-05620-0 (PMC9793633; doi:10.1186/s13071-022-05620-0)
Supplement: Supplementary file 2 — Additional file 2. Table S2: The list of small RNA sequencing data from urine in the present study. [file 13071_2022_5620_MOESM2_ESM.docx]

**Additional file 2: Table S2.** The list of small RNA sequencing data from urine in the present study.

| Library | Raw tag count | Low quality tag count | Invalid adapter tag count | PolyA tag count | Short valid length tag | Clean tag count | Q20 of clean tag (%) |
| --- | --- | --- | --- | --- | --- | --- | --- |
| Con-1 | 32,000,000 | 704,893 | 572,781 | 239 | 6,727,059 | 23,995,028 | 99.3 |
| Con-2 | 29,292,288 | 714,480 | 414,826 | 0 | 3,988,438 | 24,174,544 | 99.3 |
| Con-3 | 30,379,746 | 865,349 | 581,116 | 1 | 4,766,446 | 24,166,834 | 99.6 |
| Con-4 | 29,927,156 | 857,518 | 675,855 | 142 | 4,707,966 | 23,685,675 | 99.4 |
| AI-1 | 45,283,018 | 999,795 | 1,485,775 | 11 | 19,094,936 | 23,702,501 | 99.2 |
| AI-2 | 48,979,591 | 1,102,953 | 1,488,889 | 13 | 22,236,412 | 24,151,324 | 99.3 |
| AI-3 | 25,594,762 | 709,679 | 588,773 | 4 | 4,519,299 | 19,777,007 | 99.4 |
| AI-4 | 39,631,074 | 1,128,354 | 2,659,237 | 15 | 7,573,758 | 28,269,710 | 99.4 |
| CI-1 | 42,105,263 | 972,983 | 884,091 | 186 | 16,160,762 | 24,087,241 | 99.3 |
| CI-2 | 38,095,238 | 889,792 | 763,757 | 11 | 12,342,201 | 24,099,477 | 99.3 |
| CI-3 | 29,253,965 | 817,721 | 853,803 | 108 | 2,604,447 | 24,977,886 | 99.5 |
| CI-4 | 30,162,002 | 728,017 | 279,645 | 0 | 1,630,007 | 27,524,333 | 99.4 |

Abbreviations: Con, control group; AI, acutely infected group; CI, chronical infected group.
