# Supplementary material for: A combined miRNA–piRNA signature in the serum and urine of rabbits infected with Toxoplasma gondii oocysts
Source: Parasit Vectors. 2022 Dec 26;15:490. doi: 10.1186/s13071-022-05620-0 (PMC9793633; doi:10.1186/s13071-022-05620-0)
Supplement: Supplementary file 3 — Additional file 3. Table S3: Common dysregulated microRNAs detected in both infected serum and urine samples. [file 13071_2022_5620_MOESM3_ESM.doc]

**Additional file 3: Table S3****. Common dysregulated microRNAs detected in both infected serum and urine samples.**

|  | Serum | | | | Urine | | | |
| --- | --- | --- | --- | --- | --- | --- | --- | --- |
|  | AI vs Con | | CI vs Con | | AI vs Con | | CI vs Con | |
| miroRNA | Fold change | Q value | Fold change | Q value | Fold change | Qvalue | Fold change | Q value |
| novel_mir1 | 2.42 | 0 | 1.33 | 0 | 2.31 | 0 | -5.02 | 0 |
| novel_mir102 | -3.16 | 1.70E-96 | -1.08 | 8.88E-19 | 1.03 | 0 | -3.03 | 0 |
| novel_mir104 | -6.72 | 9.75E-13 | -5.92 | 7.96E-10 | -8.45 | 1.38E-32 | -6.09 | 1.68E-19 |
| novel_mir106 | -5.76 | 4.88E-08 | -4.96 | 3.74E-06 | -8.96 | 4.17E-42 | -6.60 | 4.34E-26 |
| novel_mir110 | -4.51 | 7.55E-05 | -3.72 | 9.43E-04 | 2.55 | 0 | -8.66 | 1.56E-84 |
| novel_mir124 | -4.83 | 1.74E-05 | -4.04 | 2.96E-04 | -8.16 | 4.50E-28 | -5.79 | 1.89E-16 |
| novel_mir13 | -8.11 | 1.37E-26 | -7.32 | 2.86E-21 | -8.19 | 1.56E-28 | -5.82 | 9.31E-17 |
| novel_mir136 | -5.68 | 9.29E-08 | -4.89 | 6.02E-06 | -8.21 | 9.21E-29 | -5.84 | 6.54E-17 |
| novel_mir139 | -5.60 | 1.79E-07 | -4.80 | 9.63E-06 | -9.32 | 1.47E-50 | -6.95 | 4.82E-32 |
| novel_mir148 | -6.37 | 1.07E-10 | -5.57 | 3.28E-08 | -9.79 | 2.71E-64 | -7.42 | 6.81E-42 |
| novel_mir149 | -6.93 | 3.09E-14 | -6.14 | 5.02E-11 | -9.19 | 2.80E-47 | -6.82 | 1.02E-29 |
| novel_mir150 | -5.51 | 3.42E-07 | -4.72 | 1.52E-05 | -9.90 | 3.99E-68 | -7.54 | 1.12E-44 |
| novel_mir152 | -6.42 | 5.92E-11 | -5.62 | 2.09E-08 | -8.98 | 1.64E-42 | -6.61 | 2.26E-26 |
| novel_mir153 | -6.55 | 9.99E-12 | -5.76 | 5.12E-09 | -9.08 | 1.01E-44 | -6.71 | 6.50E-28 |
| novel_mir156 | -6.15 | 1.18E-09 | -5.36 | 2.15E-07 | -8.78 | 2.12E-38 | -6.41 | 1.70E-23 |
| novel_mir159 | -6.09 | 2.17E-09 | -5.30 | 3.39E-07 | -8.19 | 1.56E-28 | -5.82 | 9.31E-17 |
| novel_mir161 | -6.72 | 9.75E-13 | -1.47 | 1.29E-04 | 1.40 | 3.29E-66 | 3.28 | 0 |
| novel_mir164 | -5.97 | 7.50E-09 | -5.17 | 8.91E-07 | -11.36 | 1.92E-143 | -1.60 | 1.70E-43 |
| novel_mir167 | -6.09 | 2.17E-09 | -5.30 | 3.39E-07 | -7.82 | 8.76E-24 | -5.46 | 1.34E-13 |
| novel_mir168 | -5.76 | 4.88E-08 | -4.96 | 3.74E-06 | -12.09 | 1.35E-207 | 2.75 | 0 |
| novel_mir169 | -1.99 | 3.24E-06 | 1.12 | 8.55E-05 | -9.33 | 9.58E-51 | -6.96 | 3.60E-32 |
| novel_mir17 | -5.68 | 9.29E-08 | -4.89 | 6.02E-06 | -8.28 | 6.64E-30 | -5.92 | 1.11E-17 |
| novel_mir171 | -7.87 | 2.48E-23 | -7.07 | 1.52E-18 | -8.36 | 4.97E-31 | -5.99 | 1.92E-18 |
| novel_mir172 | -5.21 | 2.32E-06 | -4.42 | 6.23E-05 | -8.12 | 1.29E-27 | -5.76 | 3.84E-16 |
| novel_mir173 | -6.03 | 4.02E-09 | -5.24 | 5.52E-07 | -3.21 | 8.24E-281 | -9.56 | 3.49E-138 |
| novel_mir177 | -5.09 | 4.36E-06 | -4.30 | 1.00E-04 | -8.14 | 7.61E-28 | -5.77 | 2.69E-16 |
| novel_mir18 | -5.42 | 6.44E-07 | -4.62 | 2.51E-05 | -8.05 | 1.13E-26 | -5.69 | 1.64E-15 |
| novel_mir184 | -6.72 | 9.75E-13 | -5.92 | 7.96E-10 | -8.89 | 1.11E-40 | -6.53 | 4.31E-25 |
| novel_mir187 | -5.09 | 4.36E-06 | -4.30 | 1.00E-04 | -9.04 | 1.02E-43 | -6.67 | 3.24E-27 |
| novel_mir189 | -5.32 | 1.22E-06 | -4.52 | 3.95E-05 | -8.87 | 2.86E-40 | -6.51 | 8.37E-25 |
| novel_mir192 | -6.60 | 5.61E-12 | -5.80 | 3.19E-09 | 1.06 | 2.93E-17 | -6.18 | 1.53E-20 |
| novel_mir195 | -5.51 | 3.42E-07 | -4.72 | 1.52E-05 | -9.05 | 4.04E-44 | -6.69 | 1.70E-27 |
| novel_mir199 | -3.84 | 5.56E-306 | -2.62 | 4.76E-164 | -10.07 | 3.01E-74 | -7.70 | 3.64E-49 |
| novel_mir20 | -7.29 | 3.79E-17 | -6.50 | 2.12E-13 | 1.05 | 0 | -4.10 | 0 |
| novel_mir200 | -5.42 | 6.44E-07 | -4.62 | 2.51E-05 | -7.96 | 1.75E-25 | -5.60 | 1.01E-14 |
| novel_mir202 | 7.67 | 7.21E-29 | 4.61 | 8.10E-05 | -9.33 | 9.58E-51 | -6.96 | 3.60E-32 |
| novel_mir204 | -5.76 | 4.88E-08 | -4.96 | 3.74E-06 | -8.85 | 7.38E-40 | -6.49 | 1.63E-24 |
| novel_mir206 | -7.26 | 6.54E-17 | -6.47 | 3.32E-13 | -9.87 | 4.36E-67 | -7.51 | 6.42E-44 |
| novel_mir21 | -8.17 | 1.95E-27 | -3.37 | 1.05E-18 | -13.52 | 0 | -11.15 | 0 |
| novel_mir210 | -5.83 | 2.59E-08 | -5.04 | 2.32E-06 | -10.09 | 4.45E-75 | -7.73 | 8.81E-50 |
| novel_mir212 | -6.68 | 1.76E-12 | -5.89 | 1.27E-09 | -8.52 | 1.09E-33 | -6.15 | 2.98E-20 |
| novel_mir213 | -5.32 | 1.22E-06 | -4.52 | 3.95E-05 | -8.02 | 3.36E-26 | -5.65 | 3.39E-15 |
| novel_mir217 | -7.09 | 1.87E-15 | -1.72 | 6.81E-06 | -8.34 | 8.34E-31 | -5.98 | 2.72E-18 |
| novel_mir23 | -5.09 | 4.36E-06 | -4.30 | 1.00E-04 | -8.36 | 4.97E-31 | -5.99 | 1.92E-18 |
| novel_mir26 | -6.42 | 5.92E-11 | -5.62 | 2.09E-08 | 2.22 | 9.26E-233 | -7.62 | 6.31E-47 |
| novel_mir27 | -8.17 | 1.95E-27 | -2.37 | 1.15E-13 | -8.12 | 1.29E-27 | -5.76 | 3.84E-16 |
| novel_mir28 | 3.95 | 1.27E-04 | 4.74 | 4.44E-05 | 2.33 | 0 | -2.88 | 0 |
| novel_mir30 | -5.76 | 4.88E-08 | -4.96 | 3.74E-06 | -8.84 | 1.18E-39 | -6.48 | 2.27E-24 |
| novel_mir31 | -1.67 | 6.95E-10 | -1.84 | 2.91E-08 | -8.92 | 2.72E-41 | -6.56 | 1.62E-25 |
| novel_mir32 | -7.18 | 3.48E-16 | -1.69 | 5.04E-06 | 2.72 | 0 | -7.48 | 3.63E-43 |
| novel_mir35 | -5.76 | 4.88E-08 | -4.96 | 3.74E-06 | 4.21 | 0 | 3.99 | 0 |
| novel_mir36 | -6.03 | 4.02E-09 | -5.24 | 5.52E-07 | -9.08 | 1.01E-44 | -6.71 | 6.50E-28 |
| novel_mir38 | -7.68 | 4.32E-21 | -6.89 | 1.10E-16 | 10.70 | 8.84E-165 | 10.07 | 5.86E-60 |
| novel_mir48 | -5.21 | 2.32E-06 | -4.42 | 6.23E-05 | -8.25 | 1.89E-29 | -5.89 | 2.24E-17 |
| novel_mir49 | -6.64 | 3.15E-12 | -5.84 | 2.03E-09 | -8.45 | 1.38E-32 | -6.09 | 1.68E-19 |
| novel_mir5 | 5.19 | 9.05E-08 | 5.19 | 4.74E-06 | -8.99 | 1.03E-42 | -6.62 | 1.64E-26 |
| novel_mir61 | -7.42 | 2.48E-18 | -1.10 | 1.06E-04 | -8.25 | 1.89E-29 | -5.89 | 2.24E-17 |
| novel_mir65 | -6.21 | 6.47E-10 | -5.42 | 1.35E-07 | -9.69 | 4.25E-61 | -7.32 | 1.41E-39 |
| novel_mir68 | -5.21 | 2.32E-06 | -4.42 | 6.23E-05 | -9.66 | 5.12E-60 | -7.29 | 8.54E-39 |
| novel_mir70 | -5.90 | 1.40E-08 | -5.11 | 1.46E-06 | -8.30 | 3.98E-30 | -5.93 | 7.86E-18 |
| novel_mir77 | -5.21 | 2.32E-06 | -4.42 | 6.23E-05 | -8.16 | 4.50E-28 | -5.79 | 1.89E-16 |
| novel_mir8 | -1.19 | 4.04E-08 | -2.93 | 1.59E-15 | -8.67 | 2.80E-36 | -6.30 | 4.99E-22 |
| novel_mir82 | 6.16 | 1.28E-12 | 4.61 | 8.10E-05 | -9.41 | 5.07E-53 | -7.05 | 8.71E-34 |
| novel_mir85 | -4.97 | 8.58E-06 | -4.17 | 1.70E-04 | -9.03 | 1.61E-43 | -6.66 | 4.45E-27 |
| novel_mir89 | -5.42 | 6.44E-07 | -4.62 | 2.51E-05 | -8.75 | 9.07E-38 | -6.38 | 4.64E-23 |
| novel_mir90 | -4.97 | 8.58E-06 | -4.17 | 1.70E-04 | -8.28 | 6.64E-30 | -5.92 | 1.11E-17 |
| novel_mir93 | -9.17 | 4.62E-47 | -1.57 | 1.86E-15 | -10.10 | 2.10E-75 | -7.74 | 5.08E-50 |
| novel_mir94 | -4.97 | 8.58E-06 | -4.17 | 1.70E-04 | -8.53 | 6.68E-34 | -6.17 | 2.14E-20 |
| ocu-let-7c-5p | -1.40 | 4.27E-213 | -1.57 | 7.45E-190 | -5.61 | 0 | -2.28 | 0 |
| ocu-let-7d-3p | -3.58 | 0 | -1.12 | 0 | -5.38 | 0 | 2.08 | 0 |
| ocu-let-7g-5p | 1.61 | 0 | -1.13 | 9.54E-273 | -6.35 | 0 | -3.05 | 0 |
| ocu-miR-125b-5p | -2.77 | 1.80E-79 | -2.02 | 7.23E-42 | -4.78 | 4.01E-125 | -4.00 | 4.54E-58 |
| ocu-miR-133a-3p | -1.86 | 1.30E-40 | 3.58 | 0 | -4.04 | 2.04E-05 | 5.06 | 2.42E-28 |
| ocu-miR-135b-5p | 1.14 | 4.76E-07 | -1.56 | 9.31E-05 | -7.56 | 9.20E-137 | -7.20 | 1.08E-70 |
| ocu-miR-138-3p | -3.59 | 3.55E-20 | -1.44 | 1.16E-06 | -5.80 | 1.68E-09 | -3.43 | 9.45E-05 |
| ocu-miR-138-5p | 3.38 | 1.31E-25 | 2.39 | 3.28E-08 | -5.52 | 1.55E-08 | -3.15 | 3.23E-04 |
| ocu-miR-155-5p | -1.56 | 1.39E-64 | -1.15 | 2.52E-31 | -9.54 | 1.55E-108 | -7.17 | 1.68E-69 |
| ocu-miR-181d-5p | -1.64 | 2.59E-10 | -2.73 | 5.94E-13 | -6.62 | 1.92E-13 | -2.25 | 4.48E-05 |
| ocu-miR-191-3p | -2.42 | 0 | -1.42 | 1.79E-214 | 2.61 | 7.74E-09 | 6.52 | 6.56E-59 |
| ocu-miR-206-3p | -1.56 | 6.16E-136 | 2.87 | 0 | -2.96 | 2.65E-08 | -2.60 | 2.04E-04 |
| ocu-miR-26b-3p | -3.15 | 2.13E-23 | -2.55 | 1.47E-14 | -4.89 | 2.75E-48 | -6.53 | 4.31E-25 |
| ocu-miR-28-3p | -1.13 | 1.58E-23 | -1.65 | 4.83E-30 | -5.62 | 7.36E-09 | 4.27 | 4.11E-44 |
| ocu-miR-31-5p | -4.97 | 8.58E-06 | -4.17 | 1.70E-04 | -5.95 | 8.92E-75 | -7.17 | 2.69E-36 |
| ocu-miR-326-3p | -2.38 | 1.88E-85 | -1.04 | 1.19E-20 | -3.80 | 9.80E-09 | -3.43 | 9.45E-05 |
| ocu-miR-34b-3p | -4.28 | 1.04E-41 | -1.11 | 5.26E-08 | -8.34 | 0 | -2.23 | 9.32E-237 |
| ocu-miR-509c-5p | 2.61 | 5.30E-12 | 2.02 | 9.73E-06 | -6.43 | 1.77E-149 | -6.23 | 1.24E-75 |
| ocu-miR-6529-5p | -1.70 | 0 | -1.23 | 0 | -2.16 | 1.94E-13 | -4.25 | 1.91E-11 |
| ocu-miR-93-5p | 1.33 | 0 | -1.06 | 4.83E-142 | -5.23 | 8.86E-195 | -5.62 | 1.19E-98 |

Abbreviations: Con, control group; AI, acutely infected group; CI, chronical infected group.
