# Supplementary material for: A combined miRNA–piRNA signature in the serum and urine of rabbits infected with Toxoplasma gondii oocysts
Source: Parasit Vectors. 2022 Dec 26;15:490. doi: 10.1186/s13071-022-05620-0 (PMC9793633; doi:10.1186/s13071-022-05620-0)
Supplement: Supplementary file 4 — Additional file 4. Table S4: Common dysregulated piRNAs detected in both infected serum and urine samples. [file 13071_2022_5620_MOESM4_ESM.doc]

**Additional file 4: Table S4.** Common dysregulated piRNAs detected in both infected serum and urine samples.

|  | Serum | | | | Urine | | | |
| --- | --- | --- | --- | --- | --- | --- | --- | --- |
|  | AI vs Con | | CI vs Con | | AI vs Con | | CI vs Con | |
|  | Fold change | Q value | Fold change | Q value | Fold change | Q value | Fold change | Q value |
| novel_pir1005 | -6.33 | 4.05E-10 | -5.64 | 3.45E-08 | 11.39 | 5.46E-210 | -6.50 | 1.90E-26 |
| novel_pir1006 | -6.70 | 4.73E-12 | -6.01 | 1.01E-09 | 9.55 | 4.41E-80 | -5.97 | 4.55E-19 |
| novel_pir1060 | -5.20 | 2.65E-06 | -4.51 | 3.08E-05 | -7.61 | 2.96E-22 | -5.35 | 6.22E-13 |
| novel_pir1078 | -5.33 | 1.32E-06 | -4.64 | 1.84E-05 | -7.69 | 3.76E-23 | -6.42 | 3.39E-25 |
| novel_pir1121 | -5.07 | 5.25E-06 | -4.38 | 5.07E-05 | 2.62 | 3.86E-83 | -5.67 | 8.15E-16 |
| novel_pir1147 | -5.65 | 1.63E-07 | -4.96 | 3.66E-06 | -7.72 | 1.36E-23 | -6.04 | 6.20E-20 |
| novel_pir1153 | -6.07 | 5.56E-09 | -5.38 | 2.67E-07 | -7.72 | 1.36E-23 | -6.29 | 3.13E-23 |
| novel_pir1180 | -7.55 | 1.45E-18 | -6.86 | 5.13E-15 | -7.72 | 1.36E-23 | -5.27 | 2.64E-12 |
| novel_pir1207 | -6.65 | 8.87E-12 | -5.96 | 1.67E-09 | -7.74 | 8.25E-24 | -7.57 | 2.65E-50 |
| novel_pir1229 | 3.58 | 2.32E-04 | 4.40 | 4.00E-05 | -7.74 | 8.25E-24 | -5.42 | 1.51E-13 |
| novel_pir1239 | -5.33 | 1.32E-06 | -4.64 | 1.84E-05 | -7.76 | 5.04E-24 | -6.97 | 2.72E-35 |
| novel_pir1246 | 5.78 | 3.32E-11 | 4.85 | 5.26E-06 | -7.78 | 3.05E-24 | -8.55 | 9.55E-89 |
| novel_pir1256 | -5.44 | 6.58E-07 | -4.76 | 1.08E-05 | -7.78 | 3.05E-24 | -7.95 | 7.72E-63 |
| novel_pir1293 | -5.74 | 8.23E-08 | -5.06 | 2.15E-06 | -7.80 | 1.85E-24 | 9.67 | 1.17E-45 |
| novel_pir1295 | 6.19 | 7.49E-14 | 6.11 | 1.63E-10 | 2.85 | 1.11E-114 | -6.02 | 1.20E-19 |
| novel_pir1325 | -6.39 | 2.15E-10 | -5.70 | 2.10E-08 | 1.73 | 1.23E-31 | -6.29 | 3.13E-23 |
| novel_pir1344 | -1.60 | 1.66E-21 | -1.99 | 1.48E-21 | -7.83 | 6.83E-25 | -6.33 | 8.59E-24 |
| novel_pir1365 | -5.20 | 2.65E-06 | -4.51 | 3.08E-05 | 1.25 | 1.73E-16 | -5.46 | 7.46E-14 |
| novel_pir139 | -5.65 | 1.63E-07 | -4.96 | 3.66E-06 | -7.85 | 4.15E-25 | -7.28 | 1.91E-42 |
| novel_pir1392 | -5.07 | 5.25E-06 | -4.38 | 5.07E-05 | -7.85 | 4.15E-25 | -5.83 | 1.87E-17 |
| novel_pir1420 | -6.20 | 1.47E-09 | -5.51 | 9.28E-08 | -7.85 | 4.15E-25 | -1.68 | 0.00E+00 |
| novel_pir1430 | -5.07 | 5.25E-06 | -4.38 | 5.07E-05 | -7.88 | 1.55E-25 | -7.30 | 8.13E-43 |
| novel_pir1435 | -4.74 | 2.66E-05 | -4.06 | 1.74E-04 | -7.88 | 1.55E-25 | -6.11 | 8.44E-21 |
| novel_pir1468 | 1.43 | 3.42E-05 | 1.58 | 4.99E-05 | -7.90 | 9.51E-26 | 2.45 | 2.43E-168 |
| novel_pir1471 | -6.83 | 7.25E-13 | -6.14 | 2.26E-10 | 1.35 | 8.88E-20 | 1.25 | 9.04E-10 |
| novel_pir1492 | -2.26 | 0.00E+00 | -3.12 | 0.00E+00 | -7.93 | 3.56E-26 | -6.91 | 7.25E-34 |
| novel_pir1524 | -5.65 | 1.63E-07 | -4.96 | 3.66E-06 | -7.95 | 2.19E-26 | -5.81 | 2.63E-17 |
| novel_pir1536 | -5.74 | 8.23E-08 | -5.06 | 2.15E-06 | -7.95 | 2.19E-26 | -6.46 | 9.40E-26 |
| novel_pir1541 | -5.55 | 3.26E-07 | -4.86 | 6.24E-06 | -7.98 | 8.43E-27 | -7.38 | 6.26E-45 |
| novel_pir157 | -5.83 | 4.22E-08 | -5.14 | 1.28E-06 | -7.99 | 5.23E-27 | -6.08 | 2.28E-20 |
| novel_pir1576 | -6.07 | 5.56E-09 | -5.38 | 2.67E-07 | -7.99 | 5.23E-27 | -5.51 | 2.61E-14 |
| novel_pir1591 | -5.99 | 1.08E-08 | -5.30 | 4.45E-07 | -7.99 | 5.23E-27 | -7.21 | 1.10E-40 |
| novel_pir1600 | -6.20 | 1.47E-09 | -5.51 | 9.28E-08 | -8.01 | 3.23E-27 | -5.40 | 2.15E-13 |
| novel_pir1660 | -5.07 | 5.25E-06 | -4.38 | 5.07E-05 | -8.01 | 3.23E-27 | -5.85 | 9.48E-18 |
| novel_pir1662 | -6.39 | 2.15E-10 | -5.70 | 2.10E-08 | -8.05 | 7.58E-28 | -5.74 | 1.44E-16 |
| novel_pir1663 | -5.65 | 1.63E-07 | -4.96 | 3.66E-06 | -8.07 | 4.70E-28 | -5.51 | 2.61E-14 |
| novel_pir1691 | -6.99 | 6.03E-14 | -6.30 | 3.09E-11 | -8.08 | 2.91E-28 | -5.84 | 1.33E-17 |
| novel_pir1697 | -5.33 | 1.32E-06 | -4.64 | 1.84E-05 | -8.10 | 1.80E-28 | -5.57 | 6.46E-15 |
| novel_pir1701 | -5.33 | 1.32E-06 | -4.64 | 1.84E-05 | -8.11 | 1.12E-28 | -5.91 | 2.45E-18 |
| novel_pir1705 | -1.12 | 1.79E-06 | -6.99 | 4.82E-16 | -8.14 | 4.35E-29 | -5.35 | 6.22E-13 |
| novel_pir1722 | 6.76 | 6.53E-19 | 5.59 | 3.45E-08 | -8.14 | 4.35E-29 | -5.71 | 2.87E-16 |
| novel_pir1729 | -5.20 | 2.65E-06 | -4.51 | 3.08E-05 | -8.15 | 2.74E-29 | -6.87 | 4.44E-33 |
| novel_pir1738 | -6.76 | 0.00E+00 | -1.35 | 0.00E+00 | -8.17 | 1.72E-29 | -6.28 | 4.31E-23 |
| novel_pir1780 | -6.44 | 1.12E-10 | -5.76 | 1.25E-08 | -8.17 | 1.72E-29 | -7.51 | 1.75E-48 |
| novel_pir1791 | -6.44 | 1.12E-10 | -5.76 | 1.25E-08 | 3.01 | 1.93E-172 | -5.61 | 3.24E-15 |
| novel_pir1808 | -5.99 | 1.08E-08 | -5.30 | 4.45E-07 | -8.19 | 6.70E-30 | -6.53 | 5.37E-27 |
| novel_pir1856 | -6.14 | 2.87E-09 | -5.45 | 1.57E-07 | -8.19 | 6.70E-30 | 2.19 | 2.10E-32 |
| novel_pir1858 | -6.55 | 3.16E-11 | -1.28 | 2.57E-04 | -8.25 | 1.03E-30 | -8.87 | 1.55E-106 |
| novel_pir1864 | -5.33 | 1.32E-06 | 1.91 | 4.45E-07 | -8.27 | 4.08E-31 | -7.85 | 3.66E-59 |
| novel_pir1868 | -5.91 | 2.15E-08 | -5.23 | 7.59E-07 | -8.31 | 1.03E-31 | -7.03 | 1.37E-36 |
| novel_pir1885 | -5.55 | 3.26E-07 | -4.86 | 6.24E-06 | -8.33 | 4.15E-32 | -7.22 | 6.18E-41 |
| novel_pir1886 | -7.14 | 5.38E-15 | -6.45 | 4.44E-12 | -8.36 | 1.67E-32 | -5.59 | 4.56E-15 |
| novel_pir1891 | -7.39 | 4.86E-17 | -6.70 | 9.46E-14 | -8.36 | 1.67E-32 | -5.65 | 1.15E-15 |
| novel_pir1895 | -5.55 | 3.26E-07 | -4.86 | 6.24E-06 | -8.37 | 1.06E-32 | -5.54 | 1.30E-14 |
| novel_pir1925 | -1.30 | 2.40E-17 | -2.11 | 2.03E-24 | -8.37 | 1.06E-32 | -6.76 | 6.03E-31 |
| novel_pir1928 | -2.02 | 5.95E-22 | -2.73 | 2.74E-23 | -8.38 | 6.77E-33 | -5.73 | 2.03E-16 |
| novel_pir1937 | -4.74 | 2.66E-05 | -4.06 | 1.74E-04 | -8.38 | 6.77E-33 | -6.25 | 1.15E-22 |
| novel_pir1948 | -5.20 | 2.65E-06 | -4.51 | 3.08E-05 | -8.39 | 4.32E-33 | -6.36 | 3.25E-24 |
| novel_pir1956 | -5.83 | 4.22E-08 | -5.14 | 1.28E-06 | -8.39 | 4.32E-33 | -7.79 | 2.63E-57 |
| novel_pir1959 | -5.44 | 6.58E-07 | -4.76 | 1.08E-05 | -8.42 | 1.76E-33 | -7.28 | 2.53E-42 |
| novel_pir1966 | -5.07 | 5.25E-06 | -4.38 | 5.07E-05 | -8.43 | 1.12E-33 | 5.22 | 0.00E+00 |
| novel_pir1968 | -6.07 | 5.56E-09 | -5.38 | 2.67E-07 | -8.45 | 4.52E-34 | -6.19 | 8.31E-22 |
| novel_pir1974 | -6.65 | 8.87E-12 | -5.96 | 1.67E-09 | -8.53 | 1.95E-35 | -6.44 | 1.79E-25 |
| novel_pir1982 | -5.65 | 1.63E-07 | -4.96 | 3.66E-06 | -8.53 | 1.95E-35 | -6.76 | 4.44E-31 |
| novel_pir1984 | -5.07 | 5.25E-06 | -4.38 | 5.07E-05 | 2.22 | 4.79E-93 | -5.64 | 1.62E-15 |
| novel_pir1988 | -6.55 | 3.16E-11 | -5.86 | 4.57E-09 | -8.54 | 1.25E-35 | -5.67 | 8.15E-16 |
| novel_pir1989 | -6.83 | 7.25E-13 | -6.14 | 2.26E-10 | -8.58 | 2.09E-36 | -7.14 | 4.97E-39 |
| novel_pir2000 | -5.07 | 5.25E-06 | -4.38 | 5.07E-05 | -8.59 | 1.35E-36 | -6.92 | 4.00E-34 |
| novel_pir2007 | -5.07 | 5.25E-06 | -4.38 | 5.07E-05 | -8.62 | 3.59E-37 | -6.42 | 3.39E-25 |
| novel_pir2014 | -5.74 | 8.23E-08 | -5.06 | 2.15E-06 | -8.62 | 3.59E-37 | -5.51 | 2.61E-14 |
| novel_pir2022 | -6.55 | 3.16E-11 | -5.86 | 4.57E-09 | -8.63 | 2.35E-37 | -5.40 | 2.15E-13 |
| novel_pir2045 | -5.91 | 2.15E-08 | -5.23 | 7.59E-07 | -8.63 | 2.35E-37 | -6.24 | 1.59E-22 |
| novel_pir2050 | -6.87 | 3.90E-13 | -6.18 | 1.40E-10 | -8.65 | 9.86E-38 | -5.38 | 3.05E-13 |
| novel_pir2064 | -5.07 | 5.25E-06 | -4.38 | 5.07E-05 | -8.67 | 4.16E-38 | 2.86 | 0.00E+00 |
| novel_pir2078 | -6.50 | 5.88E-11 | -5.81 | 7.52E-09 | -8.70 | 1.14E-38 | -6.87 | 4.44E-33 |
| novel_pir2084 | -6.50 | 5.88E-11 | -5.81 | 7.52E-09 | -8.76 | 5.59E-40 | -6.51 | 1.39E-26 |
| novel_pir2089 | -5.65 | 1.63E-07 | -4.96 | 3.66E-06 | -8.76 | 5.59E-40 | -7.21 | 1.47E-40 |
| novel_pir2090 | -5.65 | 1.63E-07 | -4.96 | 3.66E-06 | -8.76 | 5.59E-40 | -5.65 | 1.15E-15 |
| novel_pir2095 | -5.65 | 1.63E-07 | -4.96 | 3.66E-06 | -8.78 | 2.43E-40 | -6.58 | 7.91E-28 |
| novel_pir2103 | -6.70 | 4.73E-12 | -6.01 | 1.01E-09 | -8.80 | 1.04E-40 | -6.20 | 5.98E-22 |
| novel_pir2112 | 4.79 | 4.35E-103 | 3.19 | 1.69E-21 | -8.84 | 1.24E-41 | -7.69 | 3.95E-54 |
| novel_pir2114 | -5.83 | 4.22E-08 | -5.14 | 1.28E-06 | -8.85 | 8.17E-42 | 2.88 | 0.00E+00 |
| novel_pir2116 | -5.83 | 4.22E-08 | -5.14 | 1.28E-06 | -8.85 | 8.17E-42 | 2.29 | 6.20E-49 |
| novel_pir2138 | -5.83 | 4.22E-08 | -5.14 | 1.28E-06 | -8.86 | 5.42E-42 | -6.09 | 1.64E-20 |
| novel_pir2148 | -5.55 | 3.26E-07 | -4.86 | 6.24E-06 | -8.87 | 2.32E-42 | -6.59 | 5.77E-28 |
| novel_pir2188 | -6.50 | 5.88E-11 | -5.81 | 7.52E-09 | -8.92 | 1.86E-43 | 2.23 | 3.46E-130 |
| novel_pir222 | -6.99 | 6.03E-14 | -6.30 | 3.09E-11 | -8.93 | 1.22E-43 | -5.76 | 1.02E-16 |
| novel_pir2224 | -6.65 | 8.87E-12 | -5.96 | 1.67E-09 | -9.03 | 5.63E-46 | 9.77 | 3.33E-48 |
| novel_pir261 | -5.74 | 8.23E-08 | -5.06 | 2.15E-06 | -9.10 | 1.41E-47 | -5.80 | 3.69E-17 |
| novel_pir288 | 4.90 | 2.27E-07 | 4.27 | 6.39E-05 | -9.10 | 9.48E-48 | -5.38 | 3.05E-13 |
| novel_pir296 | -6.07 | 5.56E-09 | -5.38 | 2.67E-07 | -9.21 | 2.21E-50 | -6.05 | 4.44E-20 |
| novel_pir341 | -5.55 | 3.26E-07 | -4.86 | 6.24E-06 | -9.21 | 2.21E-50 | -5.83 | 1.87E-17 |
| novel_pir366 | -5.07 | 5.25E-06 | -4.38 | 5.07E-05 | -9.25 | 2.07E-51 | -5.99 | 2.35E-19 |
| novel_pir392 | -5.20 | 2.65E-06 | -4.51 | 3.08E-05 | -9.26 | 9.42E-52 | -6.03 | 8.64E-20 |
| novel_pir428 | -5.07 | 5.25E-06 | -4.38 | 5.07E-05 | -9.28 | 1.97E-52 | -6.04 | 6.20E-20 |
| novel_pir492 | -5.74 | 8.23E-08 | -5.06 | 2.15E-06 | -9.31 | 2.74E-53 | -6.20 | 5.98E-22 |
| novel_pir548 | -5.99 | 1.08E-08 | -5.30 | 4.45E-07 | -9.36 | 1.21E-54 | -6.02 | 1.20E-19 |
| novel_pir56 | -5.20 | 2.65E-06 | -4.51 | 3.08E-05 | -9.37 | 5.61E-55 | -6.42 | 3.39E-25 |
| novel_pir593 | -6.20 | 1.47E-09 | -5.51 | 9.28E-08 | -9.48 | 3.85E-58 | -6.52 | 1.01E-26 |
| novel_pir613 | -5.07 | 5.25E-06 | -4.38 | 5.07E-05 | -9.55 | 4.21E-60 | -5.85 | 9.48E-18 |
| novel_pir631 | -6.74 | 2.49E-12 | -6.06 | 6.01E-10 | -9.55 | 2.90E-60 | -5.93 | 1.25E-18 |
| novel_pir662 | -8.92 | 1.18E-38 | -1.43 | 9.52E-12 | -9.56 | 1.37E-60 | -6.19 | 8.31E-22 |
| novel_pir728 | -6.44 | 1.12E-10 | -5.76 | 1.25E-08 | 2.51 | 1.99E-270 | -6.03 | 8.64E-20 |
| novel_pir746 | -5.83 | 4.22E-08 | -5.14 | 1.28E-06 | -9.62 | 1.55E-62 | -6.69 | 9.81E-30 |
| novel_pir793 | -5.07 | 5.25E-06 | -4.38 | 5.07E-05 | -9.64 | 5.11E-63 | -5.47 | 5.26E-14 |
| novel_pir810 | -1.86 | 1.32E-12 | -1.25 | 7.51E-07 | 1.13 | 4.65E-40 | -5.51 | 2.61E-14 |
| novel_pir82 | -5.44 | 6.58E-07 | -4.76 | 1.08E-05 | 1.86 | 6.28E-142 | -5.38 | 3.05E-13 |
| novel_pir859 | -5.55 | 3.26E-07 | -4.86 | 6.24E-06 | -9.91 | 1.34E-72 | -6.31 | 1.64E-23 |
| novel_pir880 | 7.02 | 7.49E-22 | 5.75 | 7.70E-09 | -9.92 | 4.78E-73 | -6.51 | 1.39E-26 |
| novel_pir896 | -6.70 | 4.73E-12 | -6.01 | 1.01E-09 | -9.93 | 2.37E-73 | -6.28 | 4.31E-23 |
| novel_pir954 | -5.07 | 5.25E-06 | -4.38 | 5.07E-05 | 1.35 | 6.22E-73 | -5.65 | 1.15E-15 |
| novel_pir955 | -5.33 | 1.32E-06 | -4.64 | 1.84E-05 | 1.01 | 2.19E-40 | -11.37 | 0.00E+00 |
| novel_pir963 | -5.07 | 5.25E-06 | -4.38 | 5.07E-05 | -1.55 | 8.03E-41 | -5.47 | 5.26E-14 |
| novel_pir982 | -7.03 | 3.32E-14 | -2.17 | 3.94E-07 | -10.29 | 2.14E-88 | -5.44 | 1.06E-13 |
| novel_pir987 | -6.74 | 2.49E-12 | -6.06 | 6.01E-10 | -10.56 | 1.64E-101 | -5.56 | 9.17E-15 |
| novel_pir989 | -5.55 | 3.26E-07 | -4.86 | 6.24E-06 | -3.55 | 9.60E-162 | -5.44 | 1.06E-13 |
| novel_pir991 | -6.14 | 2.87E-09 | -5.45 | 1.57E-07 | -1.11 | 7.91E-49 | -7.02 | 2.48E-36 |
| novel_pir992 | -5.07 | 5.25E-06 | 1.48 | 9.83E-05 | -11.42 | 8.75E-158 | -6.94 | 1.21E-34 |
| novel_pir993 | -6.39 | 2.15E-10 | 1.53 | 9.28E-08 | -13.71 | 0.00E+00 | 3.79 | 8.60E-150 |
| novel_pir999 | -5.33 | 1.32E-06 | -4.64 | 1.84E-05 | 1.05 | 0.00E+00 | -5.80 | 3.69E-17 |

Abbreviations: Con, control group; AI, acutely infected group; CI, chronical infected group.
